# Bee products prevent VEGF-induced angiogenesis in human umbilical vein endothelial cells

**DOI:** 10.1186/1472-6882-9-45

**Published:** 2009-11-17

**Authors:** Hiroshi Izuta, Masamitsu Shimazawa, Kazuhiro Tsuruma, Yoko Araki, Satoshi Mishima, Hideaki Hara

**Affiliations:** 1 Department of Biofunctional Evaluation, Molecular Pharmacology, Gifu Pharmaceutical University, 5-6-1 Mitahora-higashi, Gifu 502-8585, Japan; 2 Nagaragawa Research Center, Api Co. Ltd., 692-3 Nagara, Gifu 502-0071, Japan

## Abstract

**Background:**

Vascular endothelial growth factor (VEGF) is a key regulator of pathogenic angiogenesis in diseases such as cancer and diabetic retinopathy. Bee products [royal jelly (RJ), bee pollen, and Chinese red propolis] from the honeybee, *Apis mellifera*, have been used as traditional health foods for centuries. The aim of this study was to investigate the anti-angiogenic effects of bee products using human umbilical vein endothelial cells (HUVECs).

**Methods:**

In an *in vitro *tube formation assay, HUVECs and fibroblast cells were incubated for 14 days with VEGF and various concentrations of bee products [RJ, ethanol extract of bee pollen, ethanol extract of Chinese red propolis and its constituent, caffeic acid phenethyl ester (CAPE)]. To clarify the mechanism of *in vitro *angiogenesis, HUVEC proliferation and migration were induced by VEGF with or without various concentrations of RJ, bee pollen, Chinese red propolis, and CAPE.

**Results:**

RJ, bee pollen, Chinese red propolis, and CAPE significantly suppressed VEGF-induced *in vitro *tube formation in the descending order: CAPE > Chinese red propolis >> bee pollen > RJ. RJ and Chinese red propolis suppressed both VEGF-induced HUVEC proliferation and migration. In contrast, bee pollen and CAPE suppressed only the proliferation.

**Conclusion:**

Among the bee products, Chinese red propolis and CAPE in particular showed strong suppressive effects against VEGF-induced angiogenesis. These findings indicate that Chinese red propolis and CAPE may have potential as preventive and therapeutic agents against angiogenesis-related human diseases.

## Background

Angiogenesis, the formation of new vessels from pre-existing endothelium, is an important process in the adult organism because it supports the increasing demands for metabolic supplies (nutrients, various growth factors, and molecular oxygen) at sites of tissue repair or regeneration, during processes such as pregnancy, the female reproductive cycle, wound healing, and revascularization of ischemic tissues. However, excessive angiogenesis (neovascularization) is also characteristic of a number of serious diseases, including cancer, rheumatoid arthritis, retinal neovascularization, and atherosclerosis. The process of capillary sprouting in any of these normal or abnormal developments is likely to involve a multitude of regulatory molecules that mediate the distinct steps of extracellular matrix remodeling, endothelial cell migration, proliferation, lumen formation, and blood vessel maturation. These angiogenic events are regulated by a wide variety of growth factors including VEGF, basic fibroblast growth factor, and hepatocyte growth factor.

Royal jelly (RJ) from the honey bee, *Apis mellifera*, is a popular traditional health food all over the world. Chemical compositional analysis has shown that RJ consists mainly of proteins, sugars, fatty acids including 10-hydroxy-2-decenoic acid (10 HDA), vitamins, and free amino acids [1]. RJ has several pharmacological functions including vasodilative activity [2], hypotensive activity [2], anti-tumor activity [3], and anti-hypercholesterolemic effects [4] among others.

Bee pollen is collected by honeybees as part of the nutrient harvest for the hive. Pollen contains carbohydrates, fatty acids, vitamins, minerals, and proteins, and is especially rich in free amino acids [5]. In traditional medicine, bee pollen is thought to be effective in prostatic conditions due to its presumed anti-inflammatory and anti-androgenic effects [6,7].

Propolis is the resinous substance collected by bees from the leaf buds and bark of trees. Chemical analysis using gas chromatography-mass spectrometry has demonstrated that approximately 150 polyphenolic compounds, including flavonoids and cinnamic acid derivatives, are present in propolis [8]. Propolis has been used in folk medicines in many regions of the world and has been reported to have various biological activities, such as anti-bacterial [9], anti-inflammatory [10], and anti-tumor effects [11]. Recently, Ahn et al. have reported that Brazilian propolis and its constituents suppress tumor-induced angiogenesis through inhibition of tube formation [12].

CAPE, a phenolic antioxidant, is included in propolis. CAPE has many biological and pharmacological effects, including anti-inflammatory [13], anti-viral [14], and anti-tumor activities [15]. CAPE is a potent and specific inhibitor of activation of the nuclear transcription factor (NF-κB) [16]. In angiogenesis, CAPE has been shown to prevent VEGF expression in CT26 colon adenocarcinoma cells [17]. CAPE also suppresses the induction of prostaglandin E 2 synthesis [18] mediated by 12-O-tetradecanoylphorbol-13-acetate and calcium ionophores. Therefore, CAPE may be a potential anti-angiogenic agent that can reduce neovascularization.

The angiostatic effects of bee products, (RJ, bee pollen, and Chinese red propolis) have not yet been extensively examined. The purpose of the present study was to investigate the effects of bee products in the control of angiogenesis. We examined the effects of RJ, bee pollen, Chinese red propolis, and CAPE against VEGF-induced tube formation, proliferation, and migration, using human umbilical vein endothelial cells (HUVECs) as a model *in vitro *system.

## Methods

### Materials

HUVECs, endothelial cell basal medium (HuMedia-EB2), human epidermal growth factor (hEGF), human fibroblast growth factor B (hFGF B), hydrocortisone, heparin, VEGF, amphotericin B, and gentamicin were purchased from Kurabo (Osaka, Japan). Fetal bovine serum (FBS) was purchased from HyClone Laboratories (South Logan, UT). Collagen type I (Cellmatrix type I-C) was purchased from Nitta Gelatin Inc. (Osaka, Japan). GM6001 was purchased from SIGMA-Aldrich (St. Louis, MO, USA). RJ, 10 HDA, bee pollen, Chinese red propolis, and CAPE were gifted by Api Co. Ltd. (Gifu, Japan). Ruboxistaurin was gifted from Sanwa Kagaku Kenkyusho Co., Ltd.

### Bee products

The RJ, produced by *Apis mellifera*, of Chinese origin, was a freeze-dried product. The bee pollen used in the present study originated from Jara pringosa (*Cistus ladanifer *L.) and Jara blanca (*Cistus albidus *L.) shrubs in Spain, and was extracted with 95% ethanol at room temperature for 24 h, and then filtrated to obtain the ethanol extract. The propolis used in this study was Chinese red propolis from Shandong, China, and was also extracted with 95% ethanol at room temperature for 24 h, and then filtered to obtain its ethanolic extract. RJ, bee pollen, Chinese red propolis, and CAPE were dissolved in dimethylsulfoxide (DMSO). DMSO, at the final concentration reached in each examination (0.1%), was added to each bee product, the non-drug control, and VEGF alone.

### HPLC analysis

The main constituents in ethanol extracts of Chinese red propolis were analyzed by high performance liquid chromatography (HPLC), the samples being injected into an HPLC system (Waters, Washington, NJ, USA) fitted with a Shim-pack CLC-ODS (Shimazu, Kyoto, Japan) C18 column (φ 6.0 ×150 mm). The mobile phase consisted of 1% acetic acid in 55% methanol. All constituents of Chinese red propolis were measured at a wavelength of 290 nm. Inject samples into HPLC system fitted with an Inertsil ODS-3 (φ 4.0 ×150 mm). The mobile phase consisted of 10 mM PBS in methanol. The constituent was measured at a wavelength of 210 nm.

### Cell culture

HUVECs were cultured in growth medium (HuMedia-EG2) at 37°C in a humidified atmosphere of 5% CO_2 _in air. The HuMedia-EG2 consists of basal medium (HuMedia-EB2) supplemented with 2% FBS, 10 ng/ml hEGF, 5 ng/ml hFGF B, 1 μg/ml hydrocortisone, 10 μg/ml heparin, 50 ng/ml amphotericin B, and 50 μg/ml gentamicin. Subconfluent monolayers of HUVECs, from passages 3 to 8, were used in the experiments.

### *In vitro *tube formation assay

An angiogenesis assay kit (Kurabo) was used according to the manufacturer's instructions. This kit consists of a 24-well cluster dish in which HUVECs and fibroblasts have been seeded in the optimal condition for capillary tube formation. The optimized angiogenesis medium in each well was changed on days 1, 4, 7, and 9 with fresh medium containing VEGF (10 ng/ml) plus various concentrations of RJ (30 to 300 μg/ml), GM6001 (10 μM), bee pollen (30 to 300 μg/ml), Chinese red propolis (0.3 to 3 μg/ml), or CAPE (1 to 10 μM). Bee products and CAPE were dissolved in DMSO and diluted with culture medium. DMSO, at the final concentration reached in each examination (0.1%), was added to the non-drug control, and samples containing VEGF alone.

On day 11, cells were fixed in 70% ethanol and stained with anti-CD31 antibody. For the evaluation of capillary tube formation (the stained tube-like structures), 100 mm^2 ^areas of each well were photographed using a CCD camera (HS all-in-one fluorescence microscope; Keyence, Osaka, Japan). These photographs were then used for measurement of the tube area (the total area of the tubes), tube length (the total length of the tubes), joints (the number of capillary connections), and paths (the number of tubes branching from the capillary-like network) of the stained tube-like structures, using angiogenesis image analyzer version 2 (Kurabo).

### *In vitro *cell proliferation assay

Subconfluent (~80%) HUVECs were trypsinized, seeded into a 96-well plate at 2000 cells/well, and incubated in HuMedia-EG2 for 24 h. The culture medium was then changed to HuMedia-EB2 with 2% FBS, and incubation allowed to proceed for 24 h. Fresh medium containing VEGF (10 ng/ml) was then added with or without various concentrations of RJ (100 to 300 μg/ml), bee pollen (30 to 300 μg/ml), Chinese red propolis (0.3 to 3 μg/ml), CAPE (1 to 10 μM), or ruboxistaurin (1 μM) and the incubation was continued for a further 72 h. Cell proliferation was estimated by measuring cell metabolic activity using a Cell Counting Kit-8 (Dojindo, Kumamoto, Japan) according to the manufacturer's instructions. The viable cell numbers were measured using a water-soluble tetrazolium salt, 2-(2-methoxy-4-nitrophenyl)-3-(4-nitrophenyl)-5-(2,4-disulfophenyl)-2H-tetrazolium (WST-8) and 1-methoxy-phenazine methosulfate. At the end of the drug treatments, the medium was replaced, then 10 μl of WST-8 assay solution was added to each well, and incubation allowed to proceed for 3 h at 37°C. Finally, the absorbance of the culture medium at 450 nm was measured using a microplate reader (Varioskan Flash, Thermo Electron Corporation, Vantaa, Finland).

### *In vitro *wounding-healing assay

An *in vitro *wound-healing assay was performed to measure unidirectional migration by HUVECs. For this, we partially modified the procedure described by Izuta et al. (2007) [19]. Briefly, HUVECs (4 × 10^4^/well) were seeded in 12-well plates coated with collagen type I, and incubated at 37°C until they attached. The HUVECs were then washed twice with PBS and incubated in Humedia-EB2 with 1% FBS for 24 h at 37°C. The monolayers of HUVECs were scratch-wounded to a 1 mm depth in a straight line using a 10-200 μl pipette-tip, and incubated with VEGF (10 ng/ml), with or without various concentrations of RJ (100 to 300 μg/ml), GM6001 (10 μM), bee pollen (100 to 300 μg/ml), Chinese red propolis (1 to 3 μg/ml), or CAPE (3 to 10 μM) for 24 h. To measure the number of endothelial cells that had migrated from the edge of the injured monolayer, images were photographed both immediately after wounding and after 24 h incubation, using a phase-contrast microscope (OLYMPUS, Tokyo, Japan). At least four points in each of three fields were examined at random for two independent wounds.

### Statistical analysis

Data are given as mean ± SEM. Statistical analysis was performed using a Dunnett's multiple-comparison test, with *P *< 0.05 being considered to indicate statistical significance.

## Results

### Bee products and their constituents

Firstly, we analyzed the content of major constituents included in RJ and Chinese red propolis. 10 HDA, a unique medium chain fatty acid, was contained 5.8% in RJ (Table 1). On the other hand, Chinese red propolis contains following constituents (caffeic acid, 1.3%; *p*-coumaric acid, 4.0%; caffeic acid phenethyl ester, 1.7%; chrysin, 5.0%; galangin, 3.7%; pinocembrin, 8.4%) (Table 1).

**Table 1:** Main component's contents in royal jelly (RJ) extract and ethanol extracts of Chinese red propolis

Royal jelly	Content (%)
• 10-hydroxy-2-decenoic acid	5.8
**Chinese red propolis**	Content (%)
• Caffeic acid	1.3
• *ρ*-Coumaric acid	4.0
• Caffeic acid phenethyl ester	1.7
• Chrysin	5.0
• Galangin	3.7
• Pinocembrin	8.4

The values represent contents of each component (%) included in RJ and Chinese red propolis.

### *In vitro *tube formation

**Figure 1:**
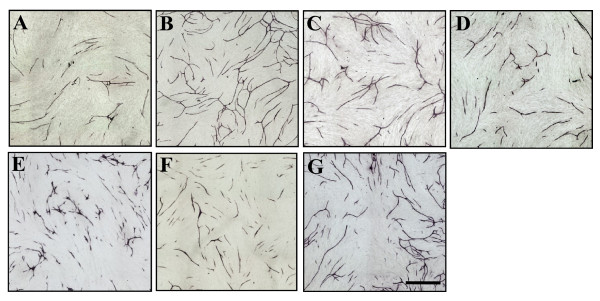
**Representative photographs of the effects of bee products on *in vitro *tube formation in HUVECs**. A) Control, B) VEGF alone, and VEGF plus C) royal jelly (300 μg/ml), D) bee pollen (100 μg/ml), E) Chinese red propolis (1.0 μg/ml), and F) caffeic acid phenethyl ester (CAPE: 3 μM), G) GM6001 (10 μM) after staining with CD-31 antibody. Scale bar represents 1 mm.

**Figure 2:**
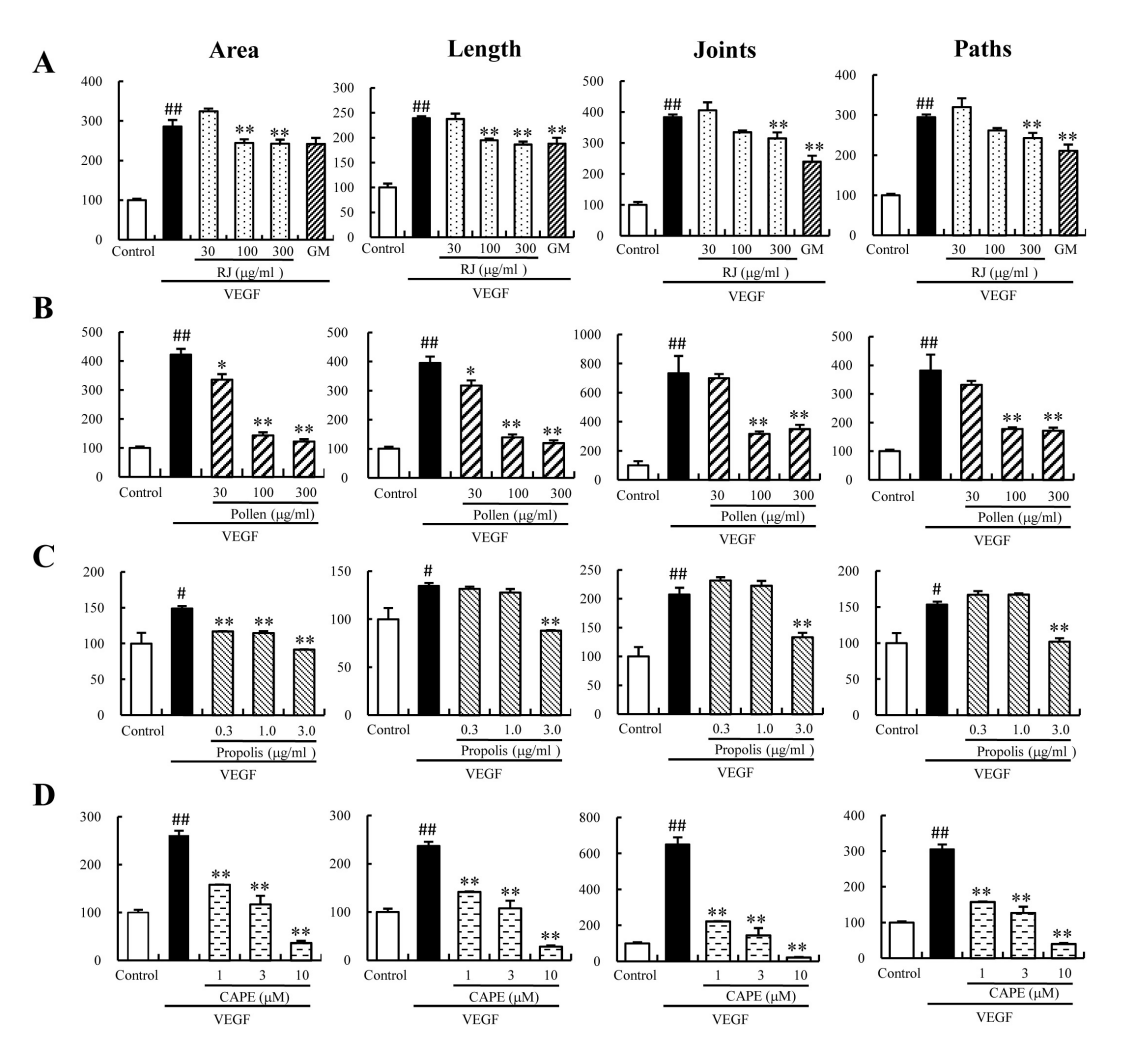
**The effects of bee products on *in vitro *tube formation in HUVECs**. A-D) Tube formation was evaluated by measurements of tube area, tube length, joints, and paths after treatment with A) royal jelly (RJ) and GM6001, B) bee pollen, C) Chinese red propolis, and D) caffeic acid phenethyl ester (CAPE), as described in "Methods". GM represents GM6001, a matrix metalloproteinase inhibitor. Data represent means ± SEM (n = 3). ##: P < 0.01 vs. Control, *: P < 0.05, **: P < 0.01 vs. VEGF alone.

New capillary formation is required for the initial steps of angiogenesis, which involves processes such as endothelial cell activation, proliferation, and migration. To investigate the inhibitory effects of bee products, we evaluated the effects of RJ, bee pollen, Chinese red propolis, and CAPE on VEGF-induced tube formation in HUVECs. VEGF stimulated the formation of capillary-like structures by HUVECs, and this action was significantly suppressed by addition of RJ, bee pollen, Chinese red propolis, and CAPE (Figure 1). To evaluate tube formation by endothelial cells in a quantitative manner, tube area, tube length, joints, and paths were measured using an imaging analyzer. RJ, bee pollen, Chinese red propolis, and CAPE suppressed the tube area following VEGF-induced tube formation. In addition, GM6001, a matrix metalloproteinase inhibitor, suppressed the tube formation. Statistically significant effects were seen for concentrations of RJ (100 to 300 μg/ml), GM6001 (10 μM), bee pollen (30 to 300 μg/ml), Chinese red propolis (0.3 to 3 μg/ml), and CAPE (1 to 10 μM) (Figure 2). These compounds also suppressed the other parameters of tube formation (tube length, joints, and paths) in a concentration-dependent manner (Figure 2).

### Cell proliferation

**Figure 3:**
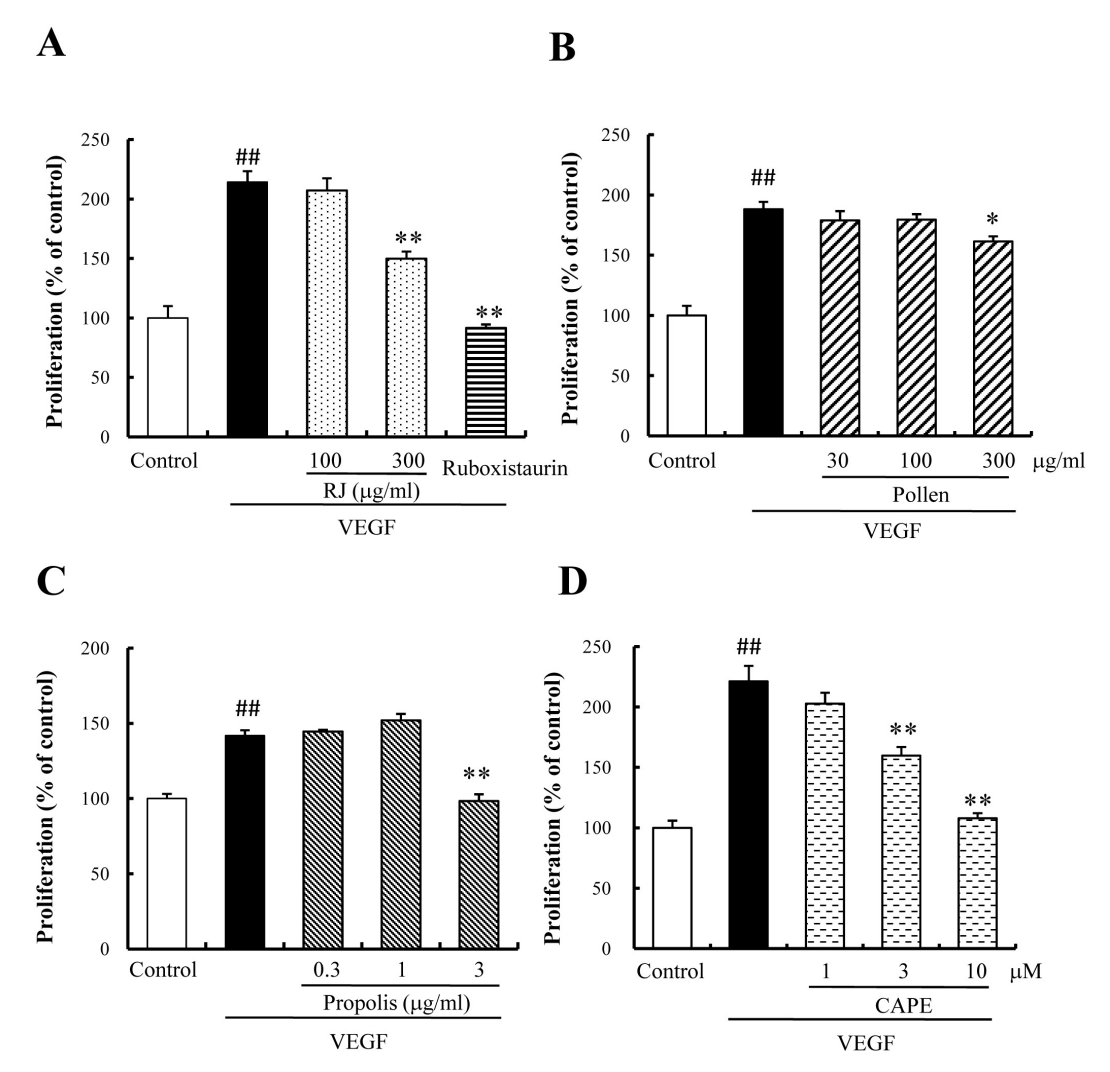
**The effects of bee products on VEGF-induced proliferation in HUVECs**. HUVECs were incubated with the indicated concentrations of (A) royal jelly (RJ) and ruboxistaurin, (B) bee pollen, (C) Chinese red propolis, and (D) caffeic acid phenethyl ester (CAPE) in the presence or absence of VEGF (10 ng/ml) for 3 days at 37°C in 5% CO_2 _with humidity. Cell proliferation was estimated using a cell counting kit-8 (CCK-8). VEGF treatments increased cell viability two-fold (vs. Control). RJ (300 μg/ml), ruboxistaurin (1 μM), bee pollen (300 μg/ml), Chinese red propolis (3 μg/ml), and CAPE (3 to 10 μM) inhibited the proliferation. Data represent means ± SEM (n = 6). ##: P < 0.01 vs. Control, *: P < 0.05, **: P < 0.01 vs. VEGF alone.

For a specific evaluation of vascular endothelial cell proliferation, a key initial step in angiogenesis, we examined whether RJ, bee pollen, Chinese red propolis, and CAPE might inhibit VEGF-induced HUVEC proliferation. In the VEGF alone group, the proliferation of HUVEC was increased 1.8-2.2 fold (vs. control). RJ, Chinese red propolis, and CAPE potently suppressed this VEGF-induced HUVEC proliferation, with effects being significant at concentrations of 300 μg/ml, 3 μg/ml, and 3-10 μM, respectively (Figure 3A, C, and 3D). On the other hand, bee pollen only weakly, but significantly, suppressed the proliferation at a concentration of 300 μg/ml (Figure 3B). In addition, ruboxistaurin, a PKC beta inhibitor, also suppressed the proliferation (Figure 3A).

### Cell migration

**Figure 4:**
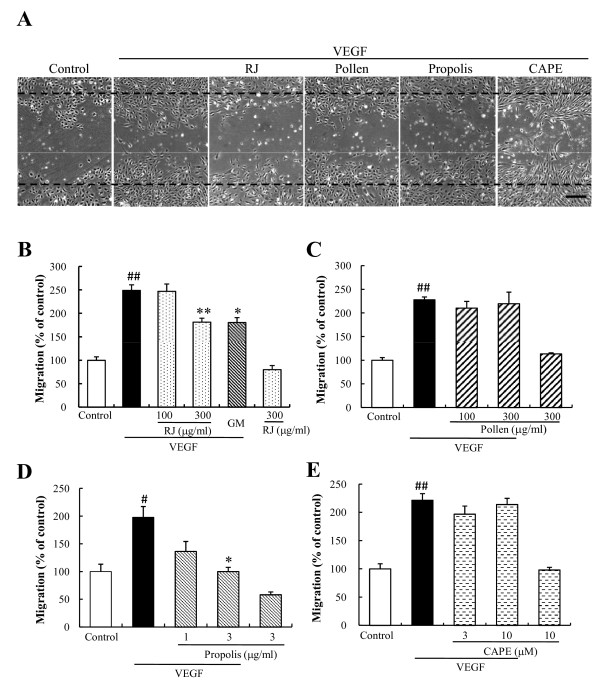
**The effects of bee products on VEGF-induced migration and *in vitro *wound healing**. A) Images of wounded monolayers of HUVEC taken at 24 h after treatment with control, VEGF alone (10 ng/ml), VEGF plus royal jelly (RJ) (300 μg/ml), VEGF plus bee pollen (300 μg/ml), VEGF plus Chinese red propolis (3 μg/ml), or VEGF plus caffeic acid phenethyl ester (CAPE) (10 μM). Scale bar represents 250 μm. Migration was estimated by measuring the cell numbers within the wounded region after treatment with VEGF (10 ng/ml) with or without (B) RJ (100 to 300 μg/ml) and GM6001 (10 μM), (C) bee pollen (100 or 300 μg/ml), (D) Chinese red propolis (1 to 3 μg/ml), or (E) CAPE (3 or 10 μM). GM represents GM6001, a matrix metalloproteinase inhibitor. Data represent means ± SEM (n = 4). ##: P < 0.01 vs. Control, **: P < 0.01 vs. VEGF alone.

For a further investigation of the anti-angiogenic effects, we tested the effects on vascular endothelial cell migration, an essential step in angiogenesis. We employed a wound-healing assay using HUVECs. Briefly, after starvation, confluent scrape-wounded HUVEC monolayers were incubated with VEGF (10 ng/ml) in the presence or absence of RJ, bee pollen, Chinese red propolis, CAPE, or GM6001, and the number of cells that had migrated into the wound region was assessed 24 h later (Figure 4A). In the VEGF alone group, the number of migrating cells was increased 1.8-2.5 fold (vs. the control group). RJ (300 μg/ml) and Chinese red propolis (3 μg/ml) significantly suppressed the VEGF-induced HUVEC migration, however bee pollen (100 to 300 μg/ml) or CAPE (3 to 10 μM) had no effect on the migration (Figure 4B-E). In addition, GM6001 (10 μM) also suppressed the migration (Figure 4B).

## Discussion

In this study, we clarified that RJ, bee pollen, Chinese red propolis, and CAPE suppressed VEGF-induced tube formation in HUVECs. The suppressive effects of Chinese red propolis and CAPE were stronger than those of RJ and bee pollen. RJ and Chinese red propolis suppressed VEGF-induced proliferation and migration in HUVECs, whereas bee pollen and CAPE suppressed only the proliferation. This is the first report of angiostatic effects of RJ and bee pollen extracts in HUVECs.

ROS production promotes angiogenesis and typical antioxidant, N-acetylcysteine suppresses VEGF-induced tube formation [20]. Therefore, ROS may play a pivotal role against VEGF-induced angiogenesis. Previously, we compared the antioxidant activities of bee products and their constituents using 1,1-diphenyl-2-picrylhydrazyl (DPPH) radical scavenging activity, and found that Chinese red propolis and CAPE exhibit strong antioxidant activities among bee products, and the IC_50 _values were 18.5 and 3.6 μg/ml (12.8 μM), whose values respectively [21]. Bee pollen extract exhibited relatively weak antioxidant activities, and the IC_50 _value was 196.7 μg/ml (Table 2) [21]. On the other hand, angiostatic activities of bee products (CAPE, Chinese red propolis, bee pollen, and RJ) exhibited 0.3 μg/ml (1 μM), 3, 100, and 300 μg/ml, whose values represented minimum concentrations suppressing all four parameters (tube area, tube length, joint, and path) in an in vitro tube formation assay. The antioxidant activities of each bee product were well corresponding with their angiostatic activities in an *in vitro *tube formation. The higher order of antioxidant activities among bee products were CAPE > Chinese red propolis >> bee pollen. On the other hand, the higher order of angiostatic activities were also CAPE > Chinese red propolis >> bee pollen. These results suggest that angiostatic effects of bee products may be partly dependent on their antioxidant activities, except for RJ.

**Table 2:** Correlation between antioxidant activities and angiostatic activities of bee products

	Antioxidant activities (μg/ml)	Angiostatic activities (μg/ml)
Caffeic acid phenethyl ester	3.6	0.3
Chinese red propolis	18.5	3.0
Bee pollen	196.7	100.0
Royal jelly	-	300.0

Antioxidant activities show the DPPH radical scavenging activities of bee products. These data represents the values of IC_50_. Angiostatic activities are represented by the minimal concentrations at which each bee products suppresses all of parameters against VEGF-induced tube formation. Data of antioxidant activities in this table were partially modified from Izuta et al. [21].

Previously, we were able to show angiostatic effect of 10 HDA, a constituent of RJ, against VEGF-induced tube formation, at concentrations of 20 μM or more [19]. In the present study, RJ suppressed VEGF-induced tube formation at concentrations of 100 μg/ml or more (Figure 2A). Dried RJ used in the present study contains 10 HDA at 5.8% (Table 1). From this composition, 10 HDA at 31.1 μM is included in RJ at a concentration of 100 μg/ml. These results indicate that the angiostatic effects of RJ may be mainly dependent on 10 HDA.

In the present study, Chinese red propolis and its constituent, CAPE, suppressed VEGF-induced angiogenesis in HUVECs (Figure 2). The minimum concentration of CAPE against VEGF-induced tube formation was 10 times lower than that of Chinese red propolis (Table 2). However, the CAPE content in the propolis was only 1.7% (Table 1), suggesting that other constituents in the propolis may influence the inhibitory effects. Previous report indicates that pinocembrin and galangin suppress tube formation in HUVECs [22], and in this study, we confirmed that these flavonoids (pinocembrin and galangin) were included in Chinese red propolis (8.4% and 3.7%, respectively). Therefore, these results indicate that not only CAPE but also other flavonoids (pinocembrin and galangin) are important components for angiostatic effect of Chiese red propolis.

In the present study, we investigated the effects of bee products on VEGF-induced cell proliferation and migration, in order to clarify the mechanism suppressing *in vitro *tube formation. RJ and Chinese red propolis inhibited VEGF-induced tube formation in a concentration-dependent manner via suppression of cell proliferation and migration of the HUVECs (Figs. 2, 3, and 4). On the other hand, CAPE inhibited the tube formation via a strong suppression of HUVEC proliferation (Figs. 2 and 3). Although bee pollen strongly inhibited *in vitro *tube formation, it exhibited only a weakly suppressive effect on cell proliferation and did not affect the migration of HUVECs (Figs. 2, 3, and 4).

In this study, to clarify the angiostatic mechanism of bee products, we examined only cell proliferation and migration assays. However, new capillary formation also involves various angiogenic processes, such as endothelial cell activation, alignment, anastomosis, and maturation of intercellular junctions. Hence, bee pollen may have a specific inhibitory mechanism on these angiogenic processes rather than on processes that regulate cell proliferation and migration.

In a previous study, we found that Brazilian green propolis and its constituent caffeoylquinic acid derivatives suppressed VEGF-induced cell proliferation, migration, and *in vitro *tube formation in HUVECs [23]. In the present study, Chinese red propolis and its chemical constituent, CAPE, suppressed VEGF-induced *in vitro *tube formation. Caffeic acid is a known suppressor of tumor angiogenesis that acts in human retinal carcinoma cells by blocking STAT3-mediated VEGF expression [24]. These studies indicate that caffeoyl groups included in propolis may be important components responsible for its anti-angiogenic activities.

The pharmacological effects of bee products have been reported in many different diseases. In particular, there are many reports about the anti-tumor effects of propolis and CAPE. Our previous studies indicate that baccharin and drupanin, constituents of Brazilian propolis, inhibit tumor growth both *in vitro *and *in vivo *[25]. Likewise, CAPE induces growth arrest and apoptosis of colon cancer cells via the beta-catenin/T-cell factor signaling pathway [26]. Tumor angiogenesis is a very important step in the growth and metastasis of tumor development. Combined with these findings in the present study, Chinese propolis and its CAPE constituent suppressed VEGF-induced angiogenesis in HUVECs indicates that the anti-tumor effects of propolis and CAPE may be dependent both on direct inhibition of tumor cell growth and on angiostatic effects on the vessels supplying nutrients to the neoplasm.

## Conclusion

Combined with the findings in the present study that Chinese propolis and its CAPE constituent suppressed VEGF-induced angiogenesis in HUVECs, the angiostatic effects of Chinese red propolis and its CAPE constituent may have potential as therapeutic agents against proangiogenic diseases.

## Competing interests

The authors declare that they have no competing interests.

## Authors' contributions

HI, MS, KT, YA-Collection, analysis, and interpretation of the data; HH-Design of the study management; MS, SM, KT, YA, HH-Preparation, review, or approval of the manuscript. All authors read and approved the final manuscript.

## Pre-publication history

The pre-publication history for this paper can be accessed here:

http://www.biomedcentral.com/1472-6882/9/45/prepub
